# Dopamine receptor autoantibody signaling in infectious sequelae differentiates movement versus neuropsychiatric disorders

**DOI:** 10.1172/jci.insight.164762

**Published:** 2024-11-08

**Authors:** Chandra M. Menendez, Jonathan Zuccolo, Susan E. Swedo, Sean Reim, Brian Richmand, Hilla Ben-Pazi, Abraham Kovoor, Madeleine W. Cunningham

**Affiliations:** 1 Department of Microbiology and Immunology, The University of Oklahoma Health Sciences Center, Oklahoma City, Oklahoma, USA.; 2 Intramural Research Program of the National Institute of Mental Health, NIH, Bethesda, Maryland, USA.; 3 Department of Pediatric Neurology, Shaare Zedek Medical Center, Faculty of Medicine, Hebrew University of Jerusalem, Jerusalem, Israel.; 4 Multidisciplinary Movement Disorders Clinic, Orthopedic Department, Assuta Ashdod, Ashdod, Israel.; 5 College of Pharmacy, University of Rhode Island, Kingston, Rhode Island, USA.

**Keywords:** Autoimmunity, Immunology, Autoimmune diseases, Neurological disorders, Psychiatric diseases

## Abstract

Despite growing recognition, neuropsychiatric diseases associated with infections are a major unsolved problem worldwide. Group A streptococcal (GAS) infections can cause autoimmune sequelae characterized by movement disorders, such as Sydenham chorea, and neuropsychiatric disorders. The molecular mechanisms underlying these diseases are not fully understood. Our previous work demonstrates that autoantibodies (AAbs) can target dopaminergic neurons and increase dopamine D2 receptor (D2R) signaling. However, AAb influence on dopamine D1 receptor (D1R) activity is underexplored. We found evidence that suggests GAS-induced cross-reactive AAbs promote autoimmune encephalitis of the basal ganglia, a region of high dopamine receptor density. Here, we report a mechanism whereby neuropsychiatric syndromes are distinguished from movement disorders by differences in D1R and D2R AAb titers, signaling, receiver operating characteristic curves, and immunoreactivity with D1R and D2R autoreactive epitopes. D1R AAb signaling was observed through patient serum AAbs and novel patient-derived monoclonal antibodies (mAbs), which induced both D1R G protein– and β-arrestin–transduced signals. Furthermore, patient AAbs and mAbs enhanced D1R signaling mechanisms mediated by the neurotransmitter dopamine. Our findings suggest that AAb-mediated D1R signaling may contribute to the pathogenesis of neuropsychiatric sequelae and inform new options for diagnosis and treatment of GAS sequelae and related disorders.

## Abstract 2

Novel human monoclonal antibody and autoantibody-mediated D1R vs D2R signaling mechanisms define basal ganglia encephalitis and neuropsychiatric infection-associated sequelae.

## Graphical abstract

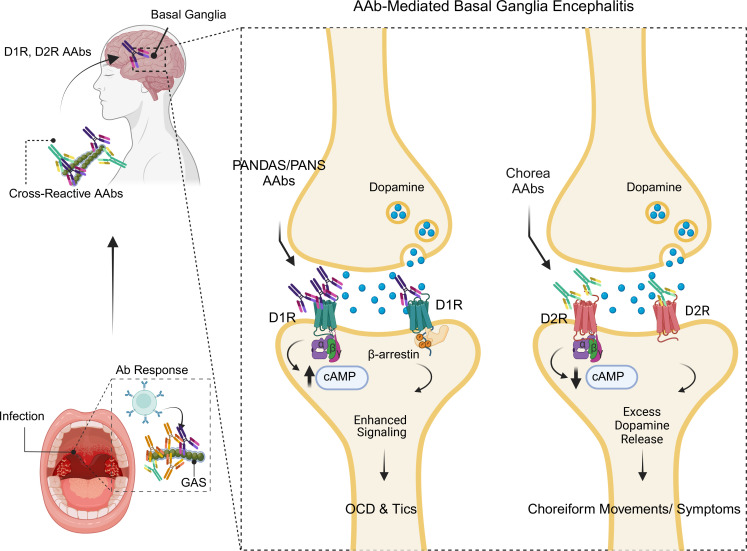

## Introduction

Movement and neuropsychiatric disorders affect millions worldwide and can be associated with microbial infections (1–8). A growing body of evidence supports the hypothesis that neuroinflammation following infections leads to autoimmune responses that target the brain (9–12). However, the pathogenic mechanisms in autoimmune neuropsychiatric diseases are complex, with few definitive biomarkers of infection-related sequelae. Pathogens such as group A streptococci (GAS) can induce autoimmune sequelae (2, 13–15), including disorders like Sydenham chorea (SC), the major neurologic manifestation of acute rheumatic fever (ARF). SC is characterized by debilitating involuntary movements and cognitive or psychotic symptoms that may develop weeks or months following a GAS infection (16–18). SC is associated with aberrant immune responses to GAS antigens that can result in cross-reactive autoantibodies (AAbs) that target the basal ganglia, including dopaminergic neurons and receptors (18–24). However, the pathogenic role and clinical importance of antibodies against the dopamine receptors in neuropsychiatric sequelae remain unknown.

The neuropsychiatric autoimmune disorder associated with GAS sequelae is described as pediatric autoimmune neuropsychiatric disorders associated with streptococcal infections (PANDAS) (25, 26). PANDAS is a heterogeneous disorder characterized by sudden onset of obsessions/compulsions or tics, and a variety of other neuropsychiatric and somatic symptoms, including anxiety, emotional lability, behavioral regression, cognitive dysfunction, disturbances of sleep, sensory perception, and micturition (25). PANDAS can be accompanied by choreiform piano playing movements of the fingers and toes, which further confounds disease classification and diagnosis (25). The pathophysiology of PANDAS is unknown, but thought to share mechanisms with SC, including infectious etiology, clinical symptoms, genetic vulnerabilities, and the potential to induce central nervous system inflammation and basal ganglia encephalitis (BGE) (16, 25, 27–29). Pathological processes that result in movement disorders or comorbid psychiatric and behavioral disorders are poorly understood (17, 30, 31). Autoimmune encephalitis is challenging to diagnose and often leads to persistent impairments in neurological, neurocognitive, and adaptive behaviors (29). Our study enhances understanding of autoimmune-mediated neurological and neuropsychiatric disease pathogenesis, categorizing subtypes of BGE associated with distinct clinical phenotypes driven by elevated titers of agonistic dopamine D1 or D2 receptor (D1R or D2R) AAbs (18, 20, 32).

Herein we build upon knowledge that D2R AAbs observed in both human disease and animal models activate signaling pathways via D2R-coupled Gi/o G proteins (20) and induce excessive dopamine release from dopaminergic neurons (33). Animal models have shown that repeated exposure to GAS infection or immunization leads to abnormal movements, repetitive behaviors, and the presence of anti-neuronal antibodies (21). Rats exposed to GAS antigens exhibit motor and behavioral changes linked to dysfunction in central dopaminergic pathways and antibody deposition in the striatum, thalamus, and frontal cortex (19, 34). In a Lewis rat model, serum AAbs targeting D1R or D2R resulted in behavioral and motor symptoms, alleviated by the D2R antagonist haloperidol (19). Expression of human-derived SC mAb V genes in Tg mouse B cells produced antineuronal AAbs in serum and targeting of dopaminergic neurons in the basal ganglia (20).

However, less is known about the role of D1R in human disease. Here, we describe the pathophysiology of D1R AAbs in neuropsychiatric sequelae associated with streptococcal infection (PANDAS). Receiver operating characteristic (ROC) curve analysis identifies D1R AAb titers’ sensitivity and specificity in autoimmune tics and obsessive-compulsive disorder (OCD), compared with D2R AAbs in choreiform movements. We characterize the first PANDAS-derived human mAbs to our knowledge and their immunological and pharmacological properties. Little is known about DR AAbs and AAb mechanisms that alter GPCR function. Our results suggest D1R AAbs enhance noncanonical signaling pathways involving G protein and β-arrestin, potentially influencing disease activity. Molecular mimicry was observed between anti–dopamine receptor AAbs and the GAS carbohydrate epitope, N-acetyl-β-D-glucosamine (GlcNAc), a cross-reactive epitope in ARF (14, 15, 35). Evidence suggests pathogenic D1R or D2R AAbs in GAS sequelae contribute to distinct clinical phenotypes. This is influenced by the prevalence of cross-reactive, somatically mutated IgG AAbs targeting D1R or D2R, which modulate neuronal signaling.

## Results

### D1R and D2R AAbs identify 2 immunophenotypes.

**Figure 1:**
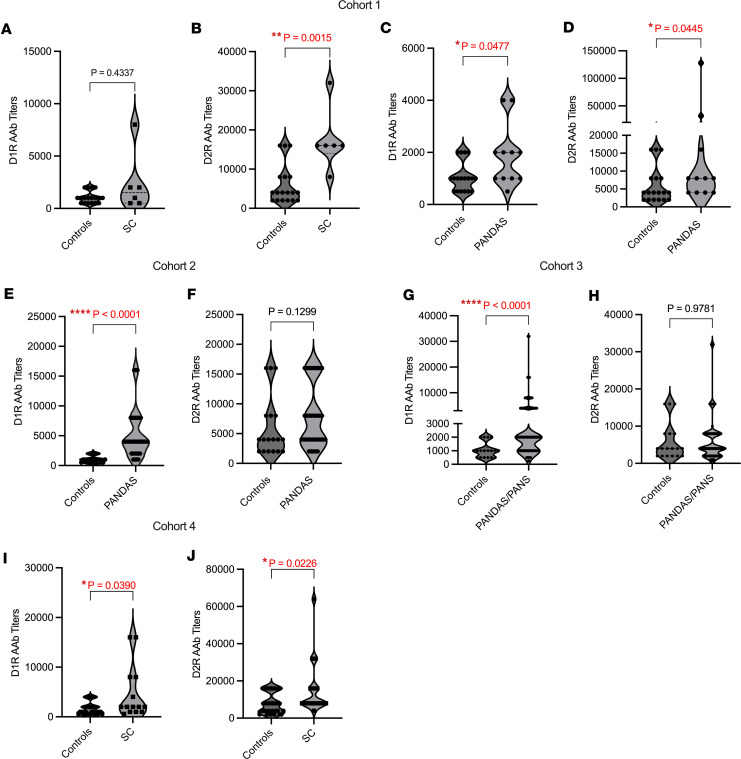
Elevated D1R and D2R AAbs in Sydenham chorea (SC) and PANDAS. (**A**–**J**) Violin plots depict AAb reactivity titrated from human sera against human D1R and D2R by ELISA from 4 cohorts. (**A** and **B**) In Cohort 1, significant differences in anti-D2R IgG levels were observed between SC patients (*n* = 6) and age-matched healthy controls (*n* = 18). (**C** and **D**) PANDAS patients with choreiform movements (*n* = 11) showed significantly higher D1R and D2R IgG AAb levels compared with controls (*n* = 18). (**E** and **F**) Cohort 2: PANDAS without choreiform movements (*n* = 35) exhibited significantly elevated anti-D1R IgG levels compared with controls (*n* = 18). (**G** and **H**) Cohort 3 with PANDAS/PANS (*n* = 858) demonstrated elevated anti-D1R AAb titer versus controls (*n* = 18). (**I** and **J**) Cohort 4: A second SC group (with acute SC) (*n* = 14) showed elevated D1R and D2R titers compared with healthy controls (*n* = 31). All groups were compared by the Mann-Whitney *U* test (*P* < 0.05 considered significant).

We analyzed serum from 1 international and 3 national cohorts to determine whether we could identify abnormally elevated AAbs targeting dopamine receptors in neuropsychiatric and movement disorders. Cohort 1 (Figure 1, A–D) included samples from the first clinical description of PANDAS who met 5 working diagnostic criteria (see Methods) in which symptom exacerbations were triggered by GAS and were studied in comparison with SC (25). Compared with age-matched healthy controls, children with PANDAS showed significantly higher AAb titers against D1R, but not in the SC group of this cohort (Figure 1, A and C). In Cohort 1, both the SC (Figure 1B) and PANDAS (Figure 1D) groups showed elevated anti-D2R AAbs when compared with healthy individuals, consistent with previous findings in children with SC (18, 20, 36). Cohort 1 revealed a significant elevation of both D1R and D2R AAbs in the PANDAS group (Figure 1, C and D). Children with PANDAS in Cohort 1 had documented choreiform piano playing movements similar to SC (25). This observation may explain abnormal elevation of D2R titers in the full-blown SC syndrome, Cohort 1 (18, 20, 36) (Figure 1B).

Cohort 2 inclusion criteria met specific PANDAS criteria, experiencing sudden onset or exacerbation of OCD 6 to 8 weeks after GAS exposure, and without recent psychoactive medication or behavioral therapy (37). This PANDAS cohort exhibited a significant elevation of anti-D1R AAbs (*P* < 0.0001) compared with healthy controls (Figure 1E), and it did not exhibit elevated anti-D2R AAbs or choreiform movements, as observed in Cohort 1 (Figure 1, C and D). Cerebrospinal fluid (CSF) from PANDAS Cohort 2, analyzed at baseline prior to the IVIG clinical trial (37), revealed 28.6% of samples positive for D1R AAbs and matched 100% of serum samples with elevated D1R AAbs (Table 1). D2R AAbs were detected in 62.8% of CSF samples, but only matched 22.8% of serum samples positive for D2R AAbs (Table 1). Although normal CSF was not available for these studies, prior assays showed no immunoreactivity with neuronal cells or human caudate putamen tissues, contrasting with positive findings in CSF and sera from PANDAS and SC patients (22, 38).

Cohort 3 included youths and young adults with OCD or tics presenting as PANDAS or pediatric acute-onset neuropsychiatric syndrome (PANS), focusing on dramatic onset or recurrence of OCD (39, 40). Although recruitment was weighted toward a history of streptococcal infections, 24.5% (211 of 858) had a documented GAS-positive test. Similar to Cohort 2, Cohort 3 sera showed a significant elevation of AAbs against D1R, but not D2R (Figure 1, G and H).

We studied a fourth group of acute SC patients from a region where rheumatic fever is endemic. This group showed significantly higher AAb titers to both D1R and D2R (*P* < 0.05) compared with healthy controls (Figure 1, I and J). These findings suggest that in some SC cases, D1R co-occurs with D2R and is linked to neuropsychiatric symptoms in SC. Immune responses against D2R in SC can persist for several weeks/months after onset (HBP and MWC, unpublished observations).

### Discriminating BGE immunophenotypes.

**Figure 2:**
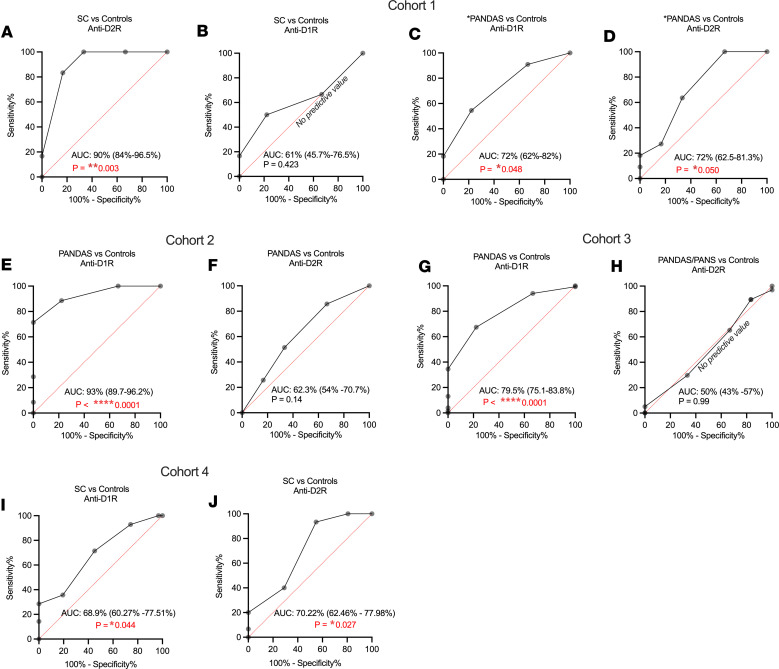
Receiver operating characteristic (ROC) curves identify anti-D1R as a sensitive and specific PANDAS/PANS biomarker and D2R for SC or PANDAS with choreiform movements. (**A** and **B**) In Cohort 1, ROC curves yield no predictive value for D1R titers and significance (*P* < 0.05 considered significant) for D2R titers in SC. (**C** and **D**) PANDAS patients with choreiform movements in Cohort 1 exhibit significant values for both D1R and D2R titers. SEM is incorporated. (**E** and **F**) Cohort 2, PANDAS without choreiform movements, demonstrates highly significant for D1R titers, with no predictive value for D2R titers. (**G** and **H**) Cohort 3 (PANDAS/PANS) also demonstrated significant AUC for D1 titers, with no predictive AUC for D2 titers. (**I** and **J**) Cohort 4/acute SC for both D1R and D2R titers separating disease from controls. ROC curve analysis with 95% confidence intervals was calculated in Prism using the Wilson-Brown method.

ROC curve analysis was used to evaluate the diagnostic accuracy of D1R and D2R AAbs as biomarkers for BGE immunophenotypes, specifically SC and PANDAS/PANS. This investigation explores whether AAbs targeting D1R or D2R can effectively identify the PANDAS and potentially the PANS immunophenotypes and distinguishing a diverse patient population with infection-associated neuropsychiatric sequelae, such as OCD and/or tics, from the SC movement phenotype. ROC curves identified SC, Cohort 1, the choreatic movement sequelae of rheumatic fever, by the presence of D2R AAbs with 90% accuracy, demonstrating robust predictive value (Figure 2A), while D1R AAbs showed no predictive value for SC (Figure 2B).

Across all cohorts, D1R AAb titers effectively discriminated PANDAS from healthy controls, confirming their role as biomarkers for PANDAS, the tic, and OCD immunophenotype of BGE (Figure 2, C, E, and G). In PANDAS Cohort 1, AUC analysis showed 72% accuracy (*P* < 0.05) for AAbs against both D1R and D2R (Figure 2, C and D), including cases with piano-playing choreiform movements. Cohort 2, a well-characterized PANDAS group without choreiform movements, demonstrated D1R AAbs with 93% accuracy (*P* < 0.0001), while D2R had no significant discriminatory capacity, 62.3% (*P* = 0.14) (Figure 2, E and F).

ROC analysis of the largest PANDAS group (PANDAS/PANS, Cohort 3) showed D1R AAbs were significantly associated with the disease at 79.5% accuracy (*P* < 0.0001), while D2R was nondiscriminatory at 50% (*P* = 0.99) (Figure 2, G and H). In Cohort 4, the SC group had predictive values for D1R and D2R AAbs at 68.9% and 70.22% accuracy, respectively (*P* < 0.05) (Figure 2, I and J). ROC analyses provide new insights, revealing the utility of DR AAbs as valuable tools in identifying BGE. Specifically, D2R AAbs characterize the SC phenotype, especially with abnormal choreiform movements, while D1R AAbs serve as a biomarker for the PANDAS/PANS phenotype.

Observing D1R or D2R AAbs in both syndromes suggests a continuum or spectrum of disease. The D1R to D2R AAb ratio correlates with symptoms (41) and may influence concomitant manifestations. The distribution of D1R AAb titers, along with sensitivity, specificity, and positive predictive rates, is shown in Supplemental Figure 1; supplemental material available online with this article; https://doi.org/10.1172/jci.insight.164762DS1

### AAbs from PANDAS and SC map to distinct D1R/D2R extracellular loop epitopes.

**Figure 3:**
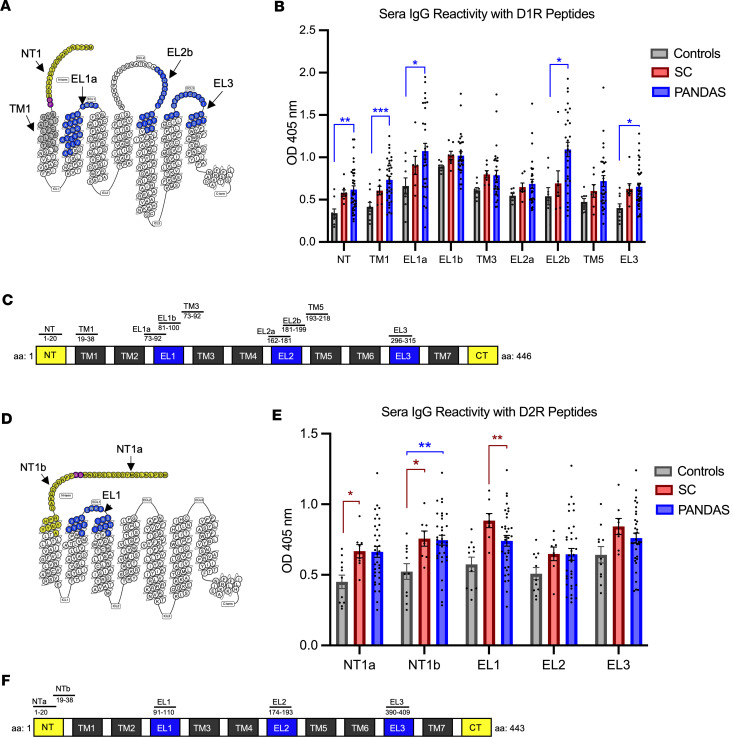
D1R and D2R AAb autoreactive epitopes mapped to distinct extracellular domains. (**A**) Diagram illustrates the D1R primary sequence for synthetic peptides that reacted with PANDAS serum IgG versus control sera. (**B**) D1R peptide reactivity in SC (*n* = 7, 8) and PANDAS (*n* = 29–36, Cohorts 1 and 2) or control sera (*n* = 8–9). One-way ANOVA with Tukey’s multiple-comparison test reveals *P < 0.05 for PANDAS (EL1a, EL2b, and EL3 epitopes) compared with control serum, and **P < 0.01, ***P < 0.001 for PANDAS (NT, TM1, or EL1a epitopes) versus controls. (**C**) Linear map of D1R and corresponding amino acid residues. (**D**) Significant D2R immunoreactive epitopes mapped by SC sera. (Overlapping peptides in purple). (**E**) D2R peptide reactivity with control (*n* = 11), SC (*n* = 8), and PANDAS sera (*n* = 34) from cohorts 1 and 2. One-way ANOVA with Tukey’s multiple-comparison test reveals greater reactivity for SC (*P < 0.05 NTa and NT1b, **P < 0.01 for EL1) versus healthy controls and PANDAS NTb (*P < 0.005) versus controls. All graphs shown as mean ± SEM. (**F**) D2R mapped linear peptides with their corresponding amino acid residues.

To determine specific immunoreactive epitopes of the dopamine GPCRs targeted by AAbs in disease, we used overlapping synthetic peptides representing human D1R and D2R extracellular and topological transmembrane domains by ELISA (Figure 3). IgG AAbs from PANDAS (Cohorts 1 and 2) recognized synthesized D1R peptide epitopes at the N-terminus (NT), the exterior of the first transmembrane domain (TM1), and had greatest reactivity with the first and second extracellular loops (EL1a, EL2b) above levels from healthy control serum (Figure 3, A and B). SC did not significantly react with D1R peptides above controls (Figure 3B). SC serum IgG primarily targeted D2R peptides (NT1a, NT1b, and EL1), exceeding reactivity from healthy serum (Figure 3, D and E), while PANDAS serum reacted with D2R NT1b (Figure 3E). Patient-serum IgG AAb recognition of D1R and D2R synthetic peptide antigenic determinants indicate that AAbs from disease predominantly target D1R or D2R extracellular epitopes. Dominant peptide specificities for D1R versus D2R peptides by PANDAS versus SC may explain differences in reactivities depicted in the ROC curves and the symptomatology of the 2 diseases. Some overlap in reactivity was anticipated between D1R and D2R in the peptide study due to sequence similarities between the receptors (Figure 8).

### AAbs against the GlcNAc epitope of the GAS group A carbohydrate antigen are elevated in PANDAS/PANS and cross-react with D1R and D2R.

Previously, we established that SC-derived human mAbs and sera bind an immunodominant epitope GAS group A carbohydrate, GlcNAc (20, 22, 35, 42). Immune responses to GAS antigens, such as the group A carbohydrate, can lead to cross-reactive AAbs in ARF (42–44). To determine whether PANDAS or PANS (PANDAS/PANS) sera have abnormally elevated antibodies against GlcNAc compared with other common microbial polysaccharides, we measured antibody titers against GlcNAc, *Haemophilus influenzae* type b capsular polysaccharide (HIB PRP), and pneumococcal polysaccharide 23F (PP-23F) antigens.

**Figure 4:**
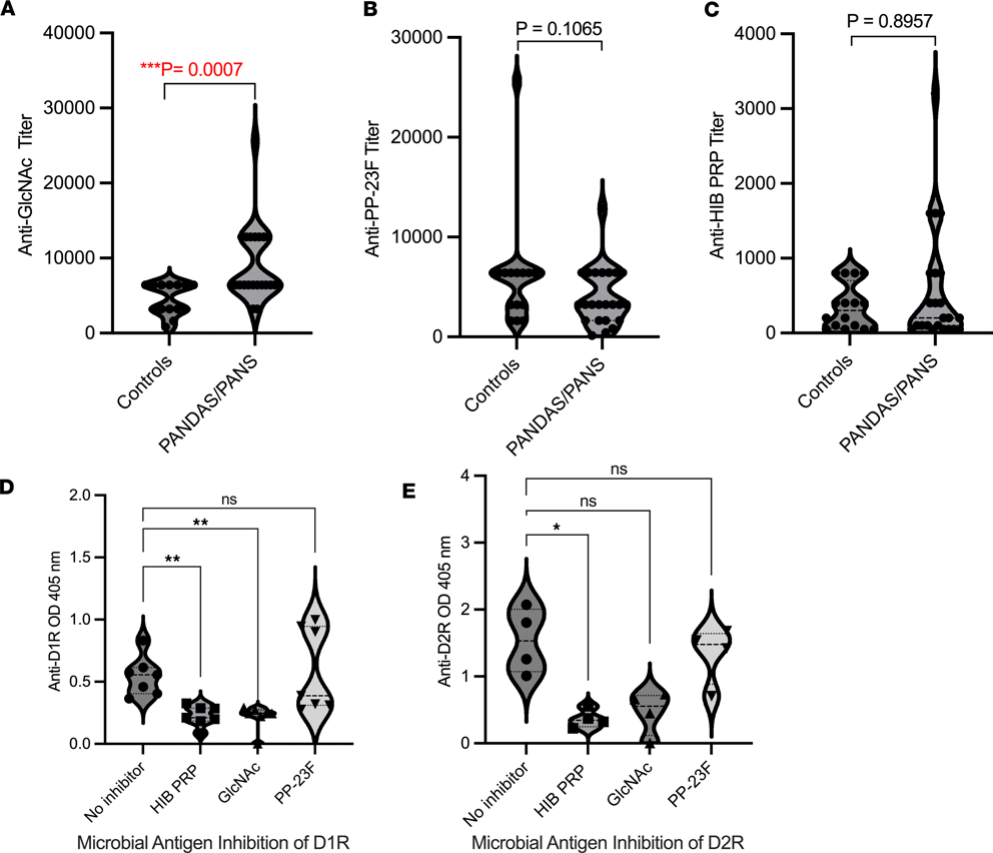
ARF GAS GlcNAc-reactive Abs are elevated in PANDAS and cross-react with D1R and HIB PRP. (**A**–**C**) ELISA titers of anti–microbial polysaccharide antigen IgG antibodies in PANDA/PANS and control serum. (**A**) Elevated anti-GlcNAc titers in PANDAS/PANS (*n* = 22) versus controls (*n* = 14). Mann-Whitney *U* test. (**B** and **C**) AAb titers against pneumococcal polysaccharide 23F (PP-23F) or *Haemophilus* *influenzae* type b capsular polyribitol phosphate (HIB PRP) were not significantly elevated (NS). (**D** and **E**) Competitive inhibition ELISA of D1R (**D**) or D2R (**E**) with PANDAS serum IgG (*n* = 7) preabsorbed with HIB-PRP, PP-23F, or GlcNAc at 500 μg/mL. GlcNAc and HIB PRP significantly inhibit PANDAS AAb IgG reactivity of D1R (**P < 0.005) and D2R (**P < 0.05). In **D** and **E**, analysis was by nonparametric Kruskal-Wallis test followed by Dunn’s multiple-comparison test.

The results show significantly elevated GlcNAc antibody titers in PANDAS serum compared with age-matched controls (Figure 4A). In contrast, titers for PP-23F and HIB PRP were not significantly elevated (Figure 4, B and C). Thus, PANDAS IgG predominantly targets the GAS carbohydrate antigen over other tested microbial antigens. Furthermore, preincubation with HIB-PRP or GlcNAc, but not PP-23F, significantly inhibited PANDAS IgG binding to D1R (Figure 4D). There was significant inhibition of HIB-PRP IgG reactivity with D2R, with a trend observed for GlcNAc (Figure 4E). Increased GlcNAc-reactive Abs in PANDAS/PANS are identified here, consistent with findings in SC (20, 22, 42, 45). Competitive inhibition of D1R and D2R AAbs by microbial antigens supports the molecular mimicry hypothesis involving microbial polysaccharide cross-reactivity with brain autoantigens D1R and D2R.

### Human PANDAS-derived mAb B4C targets EL1a of D1R, inducing dose-dependent agonistic signaling.

**Figure 5:**
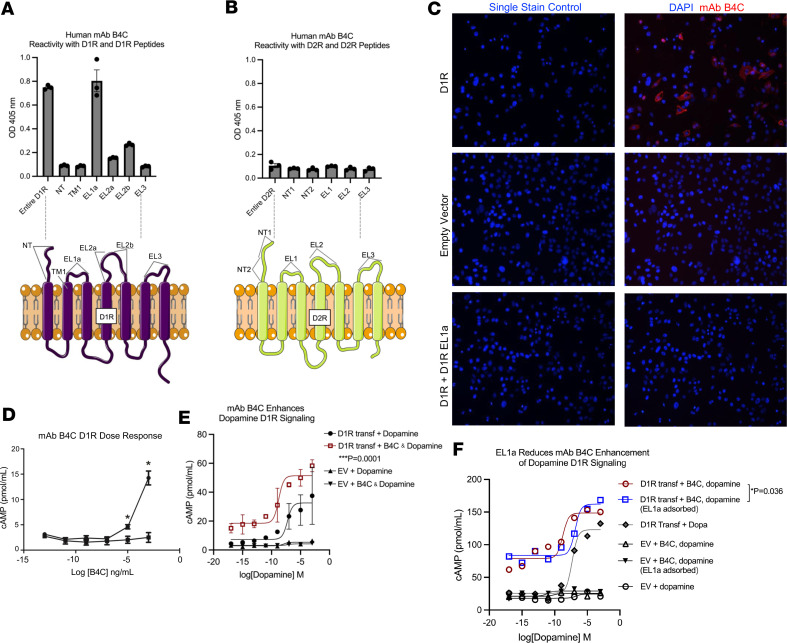
Human PANDAS–derived mAb defines specificity for D1R, targets the first extracellular loop (EL1a), signals D1R, and enhances dopamine activation of D1R. (**A**) PANDAS IgG-secreting hybridoma of B4C immunoreactivity with the synthesized human D1R and the D1R extracellular loop 1 peptide (EL1a), but not (**B**) D2R or D2R peptides. (**C**) Cell-based assay of PANDAS mAb B4C immunostaining (in red) of CHO-K1 cells transfected with D1R or the empty vector control. B4C preadsorption with the D1R EL1a peptide abolished B4C immunoreactivity with D1R. Original magnification, ×20. (**D**) mAb B4C mediates D1R dose-dependent signaling. CHO-K1 cells were transfected with full-length human D1R or the empty vector (EV) control and were treated with serial dilutions of mAb B4C starting with 1 ng/mL. *P < 0.05 by paired, 2-tailed *t* test between the D1R-transfected cells and vehicle. (**E**) CHO-K1 cells transfected with either D1R or EV were treated with mAb B4C alongside serially diluted dopamine (red) or dopamine alone (black). Dopamine dose response of D1R-transfected cells treated with dopamine versus B4C and dopamine was analyzed by nonlinear regression, showing significance. ***P = 0.0001 by extra sum-of-squares *F* test (*F* test). (**F**) CHO-K1 cells, transfected with D1R or EV, underwent treatment with serially diluted dopamine (black diamonds), mAb B4C alongside dopamine (red circles), or D1R-transfected cells with preadsorbed mAb B4C peptide EL1a (1 μg/mL) and dopamine (blue squares). Dose-response curves from the cAMP assay in D1R-transfected cells treated with mAb B4C and dopamine versus B4C and dopamine preadsorbed with D1R EL1a peptide. *P = 0.036, nonlinear regression, logEC_50_, *F* test.

A human PANDAS IgG-secreting hybridoma was developed to investigate antibody reactivity with D1R and D2R. Similar to PANDAS sera IgG, mAb B4C had specificity for D1R, but not D2R (Figure 5, A and B), contrasting with SC IgG (18, 20) and SC-derived mAbs that target D2R (20). Synthetic peptides of D1R and D2R extracellular domains verified mAb B4C’s specificity for the EL1a epitope of D1R (Figure 5, A and B). PANDAS serum IgG also recognizes D1R’s EL1a (Figure 3B), supporting mAb B4C as a representative clone of PANDAS AAbs taken from disease. mAb B4C bound D1R in transfected cells, and preadsorption with D1R EL1a eliminated mAb B4C immunolabeling of D1R, further highlighting EL1a as a critical binding site for D1R AAbs (Figure 5C).

The functional activity of PANDAS-derived human mAb B4C was evaluated by measuring intracellular cAMP induction via D1R-mediated activation of stimulatory Gαs-coupled (G_s_-coupled) G proteins in CHO-K1 cells transfected with the full-length human D1R. It was found that mAb B4C induced a dose-dependent D1R-mediated G_s_ activation (Figure 5D). Moreover, mAb B4C enhanced D1R activity beyond that induced by dopamine alone (Figure 5E). We assessed whether the D1R EL1a peptide could inhibit dopamine signaling enhancement by human mAb B4C in D1R-transfected cells compared with empty vector controls. Dopamine dose responses were measured with and without competitive inhibition by the EL1a peptide. Preadsorption with EL1a reduced mAb B4C–mediated enhancement of the dopamine response in D1R-transfected CHO-K1 cells (Figure 5F).

### AAbs from PANDAS activate D1R.

**Figure 6:**
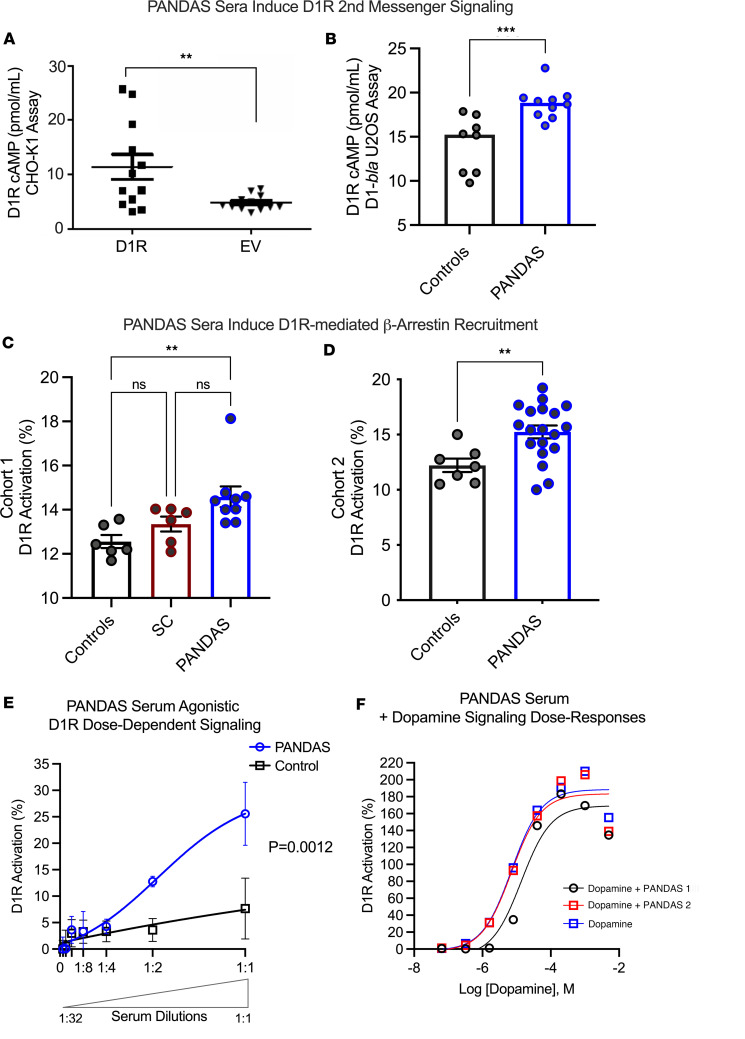
PANDAS sera activate D1R by agonist-induced increases in cAMP- and D1R-mediated β-arrestin recruitment. (**A**) PANDAS serum–induced cellular cAMP concentrations from CHO-K1 cells transfected with either D1R cDNA or the empty vector (sera diluted 1:40). **P < 0.01 by Mann-Whitney *U* test. (**B**) PANDAS sera stimulated cAMP levels in stably expressed D1R (Tango D1-*bla* U2OS cells). ***P < 0.001 by Mann-Whitney *U* test. (**C**) D1R percentage increase in β-arrestin recruitment to D1R produced by SC (*n* = 6), PANDAS (*n* = 9), and control (*n* = 6) serum from Cohort 1. **P < 0.005 controls vs. PANDAS by 1-way ANOVA with Dunn’s post hoc test. (**D**) β-Arrestin recruitment to D1R stimulated by diluted PANDAS (*n* = 19) vs. controls (*n* = 7) from Cohort 2. **P < 0.005 by Mann-Whitney *U* test. β-Arrestin recruitment to D1R was assayed using the FRET-based TANGO GeneBLAzer GPCR assay system. Values are expressed as a percentage of the FRET signal in cells relative to vehicle. (**E**) Serum-mediated D1R signaling dose response (Tango GeneBLAzer GPCR Assay) showed a significant difference between PANDAS and healthy controls (mean nonlinear regression analysis, sum-of-squares *F* test). (**F**) D1R activation is shown by the dose-response curves of PANDAS sera added in combination with dopamine (red square or blue squares) compared to dopamine alone (depicted by black circles) in Tango D1-*bla* U2OS cells.

As observed with the PANDAS-derived mAb B4C, treatment with sera from PANDAS patients also enhanced cAMP levels in D1R-transfected cells (Figure 6A) and in a GeneBLAzer cell line stably expressing D1R (Tango D1-*bla* U2OS) (Figure 6B). The increase in cellular cAMP levels reflects Gs activation by G_s_-coupled GPCRs. GPCRs can also signal through β-arrestins, distinct from canonical G protein pathways (46). PANDAS (but not SC) sera promoted β-arrestin recruitment to D1R (Figure 6C). The well-characterized PANDAS Cohort 2 (no choreiform movements) also significantly enhanced D1R activation by the β-arrestin recruitment assay (Figure 6D) and demonstrated concentration-dependent effects (Figure 6E). Combining PANDAS sera with dopamine shifted the dose-response curve of the D1R signal, trending leftward (Figure 6F). Thus, PANDAS-derived serum AAbs may enhance D1R signaling responses mediated by dopamine alone. Collectively, PANDAS-derived AAbs targeting D1R may promote both G protein–dependent and noncanonical –independent pathways.

### Autoreactive PANDAS-derived human mAbs target D1R, act as drug agonists, and enhance receptor signaling by dopamine.

**Figure 7:**
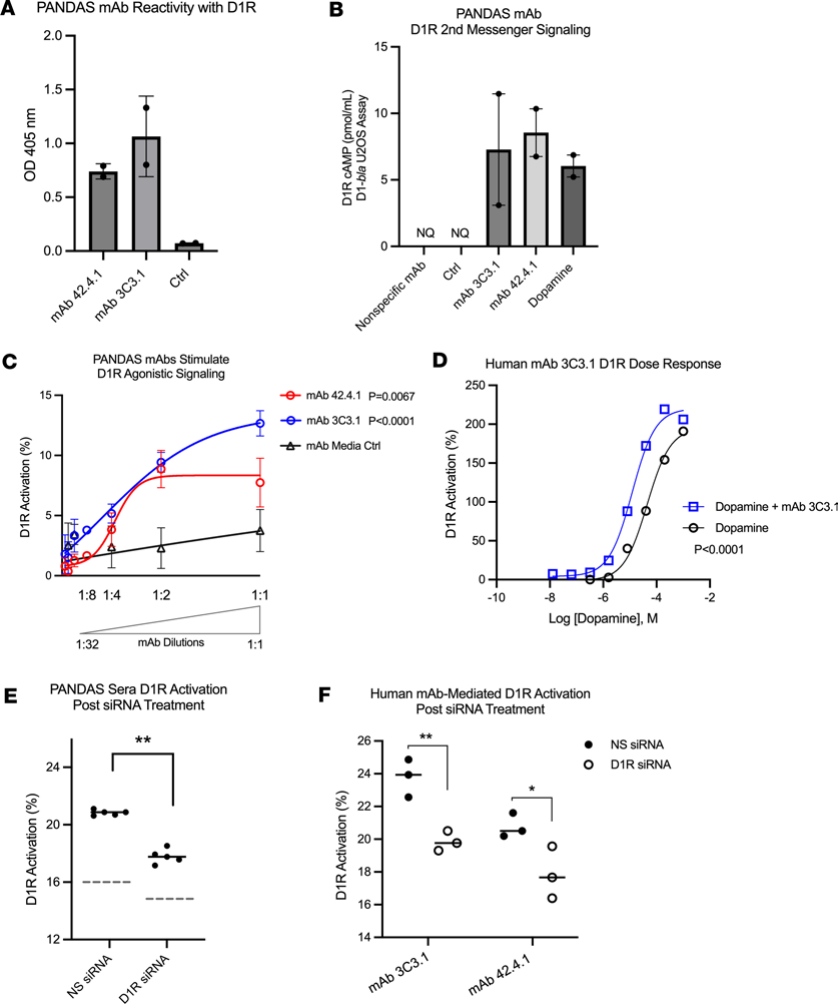
PANDAS-derived human mAbs target D1R, induce agonistic responses, and enhance receptor signaling by dopamine. (**A**) Human mAb 42.4.1 and mAb 3C3.1 reactivity with the human D1R by ELISA**.** (**B**) Human mAb 42.4.1 and mAb 3C3.1 stimulated cAMP induction in Tango D1-*bla* U2OS cells; dopamine was used at (0.001 M) as reference. Nonspecific mAb and mAb media controls were not quantifiable (NQ), as the ELISA values were below the standard curve. (**C**) Human mAbs 42.4.1 (red, **P = 0.0067) and 3C3.1 (blue, ****P < 0.0001) induced dose-dependent D1R agonistic signaling in Tango D1-*bla* U2OS cells versus media control (nonlinear regression, *F* test). (**D**) Human mAb 3C3.1 treatment with dopamine enhanced Tango D1-*bla* U2OS cells’ dopamine dose-response curve significantly, compared with dopamine alone, as per nonlinear regression, *F* test. (**E**) PANDAS sera–mediated (*n* = 5) D1R signaling of NS (nonsilencing control siRNA) or D1R siRNA–transfected D1R-*bla* U2OS. (**F**) PANDAS mAb–mediated signaling following D1R or NS siRNA transfection. In **E** and **F**, data represent the average of 3 technical replicates. *P < 0.05, **P < 0.01 by Mann-Whitney *U* test.

Two additional PANDAS patient–derived mAbs, 42.4.1 and 3C3.1, were generated to supplement studies on mAb B4C and further investigate the biological properties of autoreactive monospecific Ab clones from disease. Both PANDAS mAbs, 42.4.1 and 3C3.1, showed immunoreactivity with human D1R by ELISA (Figure 7A). Tango D1-*bla* U2OS cells treated with mAb 42.4.1 or mAb 3C3.1 resulted in AAb-mediated D1R cellular signals through the canonical activation of G_s_ proteins, as reported by elevated cellular cAMP signals (Figure 7B). Moreover, the GeneBLAzer reporter assay showed that treatment of Tango D1-*bla* U2OS cells with PANDAS-specific mAbs promoted noncanonical D1R dose–dependent enhancement of β-arrestin recruitment–mediated signaling (Figure 7C). Cotreatment of D1R-expressing cells with mAb 3C3.1 and dopamine enhanced the dose-response activity of dopamine in the assay (Figure 7D), supporting and expanding on findings with mAb B4C and AAbs from serum (Figures 5 and 6). Thus, human mAbs from PANDAS B cells, taken from individuals with the disease, mimic serum AAbs by activating D1R demonstrated by 2 distinct D1R-expressing cell lines and D1R-signaling reporter assays.

We utilized D1R-specific small interfering RNA (siRNA) to confirm that dopamine, PANDAS sera, and PANDAS-derived mAbs activate cellular signals through D1R in the Tango D1-*bla* U2OS cell line, rather than in a D1R-independent manner. D1R-targeting siRNAs substantially reduced D1R mRNA levels, confirmed by RT-qPCR (Supplemental Figure 2), and suppressed cellular responses to dopamine (Supplemental Figure 2A). Furthermore, D1R siRNA treatment attenuated the effects of PANDAS serum IgG and human PANDAS–derived mAbs 3C3.1 and 42.4.1 on D1R signaling in the GeneBLAzer assay (Figure 7, E and F).

## Discussion

Our work demonstrates specific AAb targeting of D1R by multiple PANDAS cohorts. We identify D1R as a biomarker for PANDAS/PANS and D2R as a biomarker for SC or PANDAS with choreatic movements (Supplemental Figure 3). Our new findings demonstrate that serum IgG and human-derived mAbs target the first and second extracellular loops (EL1 and -2) of D1R and induce D1R signaling in a dose-dependent manner. D1R AAbs from the PANDAS/BGE subtype sensitize D1R and enhanced dopamine-mediated signaling. D2R AAbs from SC decrease cAMP levels upon dopamine receptor activation and lead to excessive dopamine release and chorea (20, 33). Thus, we hypothesize that excess dopamine induced by AAb-mediated D2R signaling would influence D1R and sensitivity to dopamine in disease.

The current study advances understanding of neuropsychiatric sequelae associated with infections, BGE, and its global acceptance as a disease. The results help identify and delineate the poorly understood movement and neuropsychiatric disorders SC, PANDAS, and the broader disorder PANS, which can overlap clinically in neurologic and psychiatric symptoms, complicating diagnosis and treatment. Our studies of several cohorts, including the National Institute of Mental Health (NIMH) SC samples, the first clinically defined cases of PANDAS (25), the NIMH and Yale intravenous immunoglobulin (IVIG) trial (37), our large PANDAS/PANS cohort (47), and the international SC cohort demonstrate how dopamine receptors are AAb targets in 2 distinct syndromes of autoimmune BGE. The data suggest that disorders associated with GAS sequelae present as a dopamine receptor encephalitis triggered by the predominance of pathogenic autoreactive D1R and or D2R AAbs, potentially explaining acute-onset tics or OCD versus choreiform movements. The dopamine receptor AAbs identify BGE, characterize 2 BGE subtypes, PANDAS/PANS and SC, and elucidate a poorly understood pathogenic mechanism of disease, AAb signaling of GPCRs.

AAbs against D1R and D2R are among a group of anti-neuronal AAb biomarkers abnormally elevated in SC and PANDAS/PANS (27, 48, 49). Discrimination and separation of the 2 disorders have been a problem in the field for at least 20 years where PANDAS was not considered a separate entity and not fully understood as an autoimmune sequela. Studies have suggested a role for D2R in SC (18–20, 23, 36); however, its importance and frequency as a biomarker to immunophenotype BGE syndromes and its role in pathogenesis have been unclear. Our ROC analysis identified the diagnostic accuracy of AAbs in D1R as a biomarker for the clinically defined PANDAS/PANS BGE subtype in all 3 of our cohorts. In fact, anti-D1R showed the highest degree of sensitivity in the stringently characterized PANDAS (Cohort 2); it is rare to measure such a strong predictive value. Although ROC analysis predicted anti-D2R as a biomarker for SC and PANDAS when presenting with choreiform movements, there was an overall D1R AAb association with PANDAS or PANS with the tic/OCD phenotype and D2 AAbs with the choreatic movement disorders. PANDAS with choreiform movements suggests that D2R and D1R AAbs may cooperate and produce both types of disease manifestations. In support of this hypothesis, Ben-Pazi et al. reported that the ratio of D2R/D1R AAbs correlated with disease manifestations in a group of Israeli children with an SC diagnosis (41). Furthermore, highly elevated D2R AAbs in the absence of highly elevated D1R AAbs was observed in persistent chorea (chorea >1 year after onset) and suggests that D2R AAbs contribute to movement disorder manifestations. In our studies here, we used a FRET assay to demonstrate significant GPCR signaling. The signaling of D1R or D2R coincided with different manifestations, such as D1R AAbs associated with PANDAS neuropsychiatric symptoms, whereas D2R AAbs associated with SC or movement disorders. Despite the suggestive evidence for the correlation of the GPCR signaling with specific symptoms, further studies are needed to continue to understand the biologic relevance of the signaling of the dopamine receptors in neuropsychiatric diseases such as PANDAS or related streptococcal sequelae.

The data suggest that AAbs against dopamine receptors act biologically like agonists where modifying GPCR signaling mechanisms may in part be responsible for disease. GPCR AAbs are potentially cross-reactive with additional host receptors and/or with microbial antigens, as shown with a streptococcal carbohydrate epitope, GlcNAc (50) (Figure 3), and may lead to syndromes that are not well defined in human medicine (51–53). The interactions of these AAbs with their antigens may depend on susceptibility to immune responses to the original antigen, such as a microbial carbohydrate structure that cross-reacts with protein epitopes and structures in the host tissues (14, 33, 42, 45). Further maturation of the antibody repertoire may enhance cross-reactivity of the AAbs following increased exposure to infectious antigens. Shikhman et al. studied alanine replacements in GlcNAc–cross-reactive peptide sequences and demonstrated that the more hydrophobic or nonpolar residues led to cross-reactivity between host proteins and bacterial carbohydrate structures (42, 54). These types of cross-reactive immune responses develop after streptococcal and other types of infections, including SARS-CoV-2 (1, 5, 6, 18, 32, 55, 56).

Cross-reactive AAbs in GAS sequelae arise from similarities in amino acid sequences, α-helical structures, hydrophobicity, and amphipathicity between GAS carbohydrates and host antigens/receptors. This structural resemblance likely explains cross-reactivity observed between microbial antigens and GPCRs (42, 51, 52, 54, 57–59). Molecular mimicry, where immune responses target shared sequences or structures in pathogens and host receptors, may explain the cross-reactivity observed in our study. The cross-reactivities of multiple antigens recognized by antistreptococcal AAbs are attributed to protein and carbohydrate structures that mimic amphipathic or hydrophobic sequences typical of α-helical molecules found in GAS M proteins. However, the exact antigen responsible for generating these cross-reactive AAbs remains unidentified; it could involve the group A carbohydrate (GlcNAc), streptococcal M protein, or a combination of both. Disease development may result from shared immune responses implicated in all rheumatic fever and PANDAS sequelae, influenced by genetic predisposition and repeated exposure to GAS or potentially other infections with cross-reactive antigens (42, 54, 60, 61).

**Figure 8:**
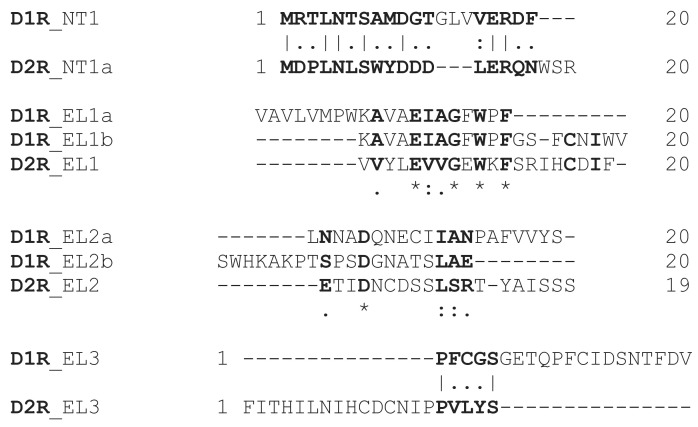
Dopamine D1R and D2R N-terminus and extracellular loop sequence alignments. Sequence alignment between the human dopamine D1R and D2R consensus sequences of similar epitopes. Identical residues are highlighted (gray). Residues with similar properties are in bold. Asterisks and/or lines indicate a single, fully conserved residue. A colon indicates conservation of similar properties. A period indicates conservation of weakly similar properties. Emboss pairwise alignments (https://www.ebi.ac.uk/jdispatcher/psa/emboss_needle) were used for 2 sequences and Clustal Omega (https://www.ebi.ac.uk/jdispatcher/msa/clustalo) for multiple sequence alignments.

Overlapping sequences were observed between D1R and D2R immunoreactive epitopes (Figure 8). Antibody cross-reactivity between these receptors may explain overlapping symptomatology in neuropsychiatric sequelae, such as acute tics or OCD associated with predominant D1R AAbs, and choreiform movements with elevated D2R AAbs (18, 20, 36). Overall antibody specificity could also account for the involuntary movements observed in SC, which is more associated with D2R AAbs (18, 20, 36), and has been effectively treated with haloperidol, a D2 blocker (19, 62). These diseases typically present as distinct entities, where one is predominantly tics and OCD in PANDAS/PANS, and the other is a movement disorder/chorea. Whether historical reports of “rheumatic psychoses” by Dr. Sydenham could have been PANDAS is uncertain.

We are the first to our knowledge to map the epitope specificity of human autoreactive basal ganglia Abs from SC and PANDAS with D1R and D2R topological and EL peptides. PANDAS and SC serum IgG show similarities in reactivity toward D1R and D2R peptides, although some peptides show specificity for either PANDAS or SC IgG. PANDAS serum IgG predominantly targets EL1a and EL2b peptides, substantiating findings from the mAb B4C clone, which also recognized D1R EL1a as an epitope targeted by disease-related AAbs. PANDAS serum IgG also reacts significantly with NT, TM1, and EL3 peptides of D1R (and NT1b of D2R), whereas SC serum IgG targeted the EL1 and NT epitopes of D2R above healthy control levels (Figure 3). Similarity in peptide reactivities between SC and PANDAS serum IgG AAbs is expected due to shared amino acid sequence homologies and structural similarities of D1R and D2R as GPCR antigens, characterized by similar hydrophobicity and amphipathicity (57) (Figure 8). However, distinctions exist in the reactivity patterns, where PANDAS IgG reacts with D1R and chorea IgG reacts with D2R, suggesting separation between the diseases. Conformational epitopes, which are challenging to assess, likely contribute to the overall reactivity and signaling function of these AAbs.

The synthesized peptides represent small receptor fragments recognized by serum Abs from disease, with varying specificities due to the heterogeneous mixture of IgG AAbs in serum. Collectively in serum, these AAbs may exhibit heightened avidity for the intact receptor structure, potentially contributing to symptomatic variations between PANDAS and SC, as illustrated in our hypothetical model (Supplemental Figure 3). Advanced studies on how AAbs bind and activate GPCRs according to their conformational properties are yet to be addressed. While the number of samples in our study was limited to those conventionally identified by us and collaborators, it became evident that SC and PANDAS exhibited similarities but could be differentiated by Ab specificities for D1R versus D2R and by particular EL epitopes. This distinction aligns with previous findings, which highlight differences between D1R and D2R despite their sequence similarities as GPCRs (Figure 8) (57).

Our studies highlight that PANDAS and SC AAbs have similar properties to pathophysiological AAbs that target GPCRs’ first and second ELs in other forms of autoimmune disease (52, 53, 63, 64). β_1_/β_2_-Adrenergic receptor and M2 acetylcholine receptor AAbs are found in patients with Chagas disease (58), Graves disease (59), and in orthostatic hypotension and tachycardia (52, 53). However, studies remain in the early stages of understanding how AAbs potentially affect GPCRs and cause diseases such as cardiomyopathy (65), myocarditis (51), glaucoma (64), and now BGE (20). Our work is the first to our knowledge to study AAb GPCR signaling effects using cell lines expressing human D1R, while also demonstrating that siRNA specific for D1R will eliminate the IgG/human mAb–mediated signaling. This evidence of specificity for D1R together with the large cohorts and epitope mapping supports the hypothesis that D1R is the Ab target in these neuropsychiatric infectious sequelae.

Abs are practical biomarkers, as they are relatively stable and easy to measure from biospecimens. While AAb biomarkers may not be directly causative in autoimmune disease or encephalitis (66), D1R and D2R AAb biomarkers provide diagnostic value and contribute to an understanding of biological AAb-mediated dopamine receptor signaling mechanisms in disease that associate with symptoms (18, 19, 27, 41, 48, 49, 67). These AAbs access the brain based on clinical findings and animal studies where immunization with streptococcal antigens cause induction of the AAbs against D1R and D2R, as suggested by Western blotting and detection in the CSF (19, 20, 22–24, 38, 48). Human SC mAbs (22) expressed in Tg mice (20) and anti-GAS IgG penetrate the BBB, and target the basal ganglia (3, 19, 20, 23, 24, 48). Creating a Tg mouse model similar to the human D2R–Tg mouse model expressing SC human mAb 24.3.1 V genes (20, 22) is impractical for this study. However, future research could adopt this strategy with PANDAS human mAb V genes to enhance understanding of AAb localization and behavioral correlations. Notably, mAbs have already enhanced understanding of these diseases and may elucidate how PANDAS patient IgG targets and affects cholinergic interneurons recently identified as AAb targets (31, 68).

Upper respiratory tract infections activate immune cells in the nasopharyngeal mucosa and induce cytokines that disrupt the blood-brain barrier, contributing to postinfectious autoimmune pathogenesis (3, 14, 69). These findings support the role of infections with cross-reactive immunity in disease, potentially enabling anti–dopamine receptor AAbs to trigger brain inflammation or pathogenesis in BGE. The results underscore immunomodulatory options for more targeted therapies where IVIG and plasma exchange have been effective in clinical trials (37, 70, 71), including successful treatments for SC aimed at reducing immune responses (72, 73).

The role of underlying mechanisms of cross-reactive immunity to microbial antigens in neuropsychiatric sequelae or BGE is highlighted by Figure 3 where anti-microbial Abs are significantly elevated against GlcNAc, an important immunoreactive antigen in ARF pathogenesis where cross-reactive AAbs target host neuronal antigens (20, 22, 50, 74). Furthermore, the molecular mimicry hypothesis whereby microbes share antigens with host tissues such as the brain and lead to autoimmune sequelae is supported by our data in that microbial antigens were sufficient to remove AAb reactivity with D1R and D2R (Figure 4). Importantly, our data suggest the GAS carbohydrate antigen GlcNAc cross-reacts with D1R and D2R AAbs from patients with disease. Although mimicry and cross-reactivity are important in defining AAbs in BGE (48), selective recognition of D2R in SC and D1R in PANDAS/PANS delineates these diseases. These AAbs may be important in defining symptoms and responsible for the correlation with the UFMG SC Rating Scale (USCRS) score of SC patients (41). Furthermore, D1R and D2R AAbs are much more predictive of BGE than GAS diagnostic antibody assays for anti–streptolysin O (ASO) or anti–DNase B, which cannot be detected in approximately 40% of pharyngitis cases (48). Identification of D1R or D2R AAbs supports treatment with immunomodulatory therapies to remove or suppress the generation of AAbs when severe symptoms warrant treatment (70).

Importantly, our study reveals that D1R AAbs are associated with neuropsychiatric symptoms observed in PANDAS or PANS (with predominant tics and OCD), whereas D2R AAbs are linked to streptococcal sequelae and movement symptoms, such as those seen in SC. The ratios of the AAbs correlate with symptoms may contribute to the hybrid manifestations of disease (41). The inability of Dale and Brilot (18) to detect D1R in SC or PANDAS samples may be due to lower sensitivity of cell-based assays or FACS analysis, or by limited D1R expression on their transfected cell lines. Their patient cohort might have lacked statistical power to detect D1R AAbs, or they could have studied sera without detectable D1R AAbs. Therefore, the present study adds clarity for understanding of these 2 disorders and the 2 dopamine receptor antigens.

Studies conducted in an international cohort of SC patients identified the presence of D1R and D2R AAbs for approximately 2 weeks in children with streptococcal pharyngitis. However, in pharyngitis, these antibodies rapidly disappeared during recovery. In contrast, in SC, an autoimmune condition, the D1R and D2R AAbs persisted and D2R AAbs progressively increased with persistent chorea (HBP and MWC, unpublished observations).

In further investigation of AAbs in healthy individuals, previous studies have included AAbs from B cell hybridomas derived from human tonsils after tonsillectomy, alongside V gene sequencing of AAbs from human and mouse hybridomas. Sequence and avidity analyses indicated that high-avidity cross-reactive anti-streptococcal/anti-heart AAbs were most damaging and cytotoxic (75). In contrast, less avid anti-streptococcal AAbs from the normal human B cell repertoire, with fewer CDR region mutations, did not recognize cell surface antigens (76–78).

Recent research has defined potential underlying mechanisms in the GAS sequelae. IgG subclass IgG2, specific for the GlcNAc epitope, may represent a previously unrecognized biomarker in ARF, recognizing human heart and neuronal cell surface antigens (50). Other mechanisms, such as defects in T follicular cells and activation by repeated streptococcal infections may lead to the emergence of more avid clones with stronger antigen specificity (56, 79). Th1 and Th17 responses in the nasal and respiratory mucosa, along with compromised blood-brain barrier permeability, may enhance AAb transfer into the brain in both SC and PANDAS. This observation has been demonstrated at the olfactory bulb following intranasal GAS infection (3, 69). The presence of B cells in the CSF may explain the enhanced level of anti-neuronal AAbs or IgG2 GlcNAc-reactive AAbs in the CSF (48).

Our collective findings that PANDAS AAbs target D1R at its ELs resulting in the enhancement of dopaminergic signaling in the presence of dopamine suggest that AAbs can allosterically activate D1R above that of dopamine alone. PANDAS-derived human mAbs, including mAb B4C, mAb 42.4.1, and mAb 3C3.1, bind and activate D1R, potentially acting as allosteric drug activators to enhance the effects of dopamine-induced D1R signaling. Preliminary evidence suggests these AAbs may serve as unique selective agonists like drugs that induce (D1R) or inhibit (D2R) distinct biological signal transduction pathways leading to excess dopamine or receptor signaling. Not only are these observations valuable to inform therapy to reduce dopamine receptor signaling in disease, but also important in the identification of Ab-accessible GPCR switches, distinct from the binding site of endogenous ligands that could activate desirable subsets of GPCR signaling pathways. Thus, our findings may be valuable for future GPCR drug discovery and for understanding the allosteric behavior of AAbs targeting GPCRs.

Our work herein advances the understanding and characterization of autoimmune BGE, SC, and PANDAS or PANS based on AAbs to dopamine receptors and identifies potential AAb mechanisms that could lead to behavioral and neuropsychiatric dysfunction. AAbs that target D1R lead to dopaminergic signaling abnormalities by enhancement of GPCR activity, while D2R AAbs reduce GPCR activity yet lead to excess dopamine release (20, 33, 36, 38, 49) (Supplemental Figure 3). Identification of D1R or D2R AAbs as biomarkers of PANDAS and SC, respectively, is fundamental for understanding treatment options, including dopamine receptor–specific antagonists, therapy to reduce AAbs, or targeted B cell or other immunomodulatory therapies. Our evidence further supports the hypothesis that in PANDAS and SC, AAbs signal dopaminergic GPCRs and lead to the pathogenesis of neuropsychiatric and movement disorders of the brain and beyond.

## Methods

### Sex as a biological variable.

Our study examined both male and female participants.

### Study participants.

In Cohort 1, SC patients were diagnosed according to the Jones Criteria, and PANDAS patients were diagnosed at the NIMH (25). Criteria included OCD and/or tic disorder, pediatric onset, episodic symptom severity, association with GAS infection, and neurological abnormalities. SC individuals were identified by 2 independent neurologists specialized in movement disorders. PANDAS children were excluded if they had SC, rheumatic fever, another autoimmune disorder, or cardiac abnormalities (25). Cohort 2 included baseline samples from the NIMH-Yale IVIG trial for PANDAS (37). Participants met PANDAS diagnostic criteria, had moderate-severe OCD symptoms (CY-BOCS score ≥20), and were not on psychoactive medications or therapy for 6 weeks prior. They had symptom onset within 6 months of a GAS infection, sudden onset or exacerbation of OCD, and 3 or more comorbid neuropsychiatric symptoms (37). Cohort 3 included individuals from the University of Oklahoma Health Sciences Center (OUHSC) with tic disorder and/or OCD suspected to have PANDAS or PANS (25, 47). Recruitment prioritized individuals with a detailed history of streptococcal infection, confirmed by either positive or negative cultures or anti-streptococcal Ab titers (ASO or anti–DNase B) (47). Two hundred and eleven of the 864 patient volunteers had a documented case of streptococcal infection at the time of sample collection. Participants on psychotropic medication, antibiotics, or steroids for their condition were not excluded. Additionally, patients who had previously received IVIG or plasma exchange in the past but were experiencing symptoms were not excluded. Healthy controls had normal physical examination findings; no lifetime personal history for the participant or any first degree relative with a Diagnostic and Statistical Manual of Mental Disorders, 4th ed. (DSM IV) diagnosis of a tic disorder, Tourette syndrome, OCD, or attention-deficit/hyperactivity disorder (ADHD); and had ASO titers ranging between 70 and 513 (Todd units) (80). Cohort 4 included patients enrolled with acute SC (*n* = 31, age 10 ± 2.7 years) and healthy children with no history of pharyngitis in the past 6 months (*n* = 31, age 14.27 ± 5.29 years). All cohorts followed IRB protocols and included healthy participants enrolled in research protocols from the NIMH or the Yale Child Study Center. Healthy controls had normal physical examination findings; no lifetime personal history for the participant or any first-degree relative with a DSM IV diagnosis of a tic disorder, Tourette syndrome, OCD, or ADHD; and had ASO titers ranging between 70 and 513 (Todd units) (80).

### Study procedures and approval.

Our study utilized participant blood and data approved by IRBs at the OUHSC and NIMH in Bethesda, Maryland, and Yale Child Study Center in New Haven, Connecticut (25, 37). Participants were recruited nationwide via NIMH website postings and ClinicalTrials.gov (NCT01281969) (Cohort 2), as well as clinician referrals. Parents or legal guardians provided written consent, and participants’ assent was given for youths 7 years of age or older, and when age appropriate (≥13 years), participants gave written assent (37). For Cohort 4, the study was approved by the Shaare Zedek Medical Center Helsinki committee and OUHSC IRB and registered at ClinicalTrials.gov and OUHSC IRB, registered at ClinicalTrials.gov (both NCT04084977). Clinical data on sex, age, symptom duration, and medical treatments were recorded. Blood samples were collected nationwide and returned overnight to the research laboratory (47). Serum samples were stored at –80°C upon arrival at OUHSC on dry ice.

### ELISA.

ELISAs followed established protocols (20, 22, 48). Briefly, Immunolon 4 microtiter plates (VWR) were coated overnight at 4°C with 10 μg/mL of antigens: D1R (Perkin-Elmer), D2R (Perkin-Elmer or in-house), GlcNAc conjugated to BSA (Vector Labs), HIB PRP (National Institute for Biological Standards and Controls, London, United Kingdom), pneumococcal capsular antigens (*Streptococcus pneumoniae* type 23F, ATCC), or D1R and D2R synthesized peptides (for peptide epitope mapping) (Table 2) in carbonate/bicarbonate buffer pH 9.6. Sera or CSF was serially diluted in 1% BSA/PBS (Thermo Fisher Scientific) and incubated overnight at 4°C. Plates were washed 5 times with 0.05% Tween 20. Alkaline phosphatase–conjugated anti–human IgG secondary antibody (1:1000; Sigma-Aldrich) was added in 1% BSA/PBS. Antibody titers represent the serum dilution giving an optical density of 0.1 at 405 nm after 2 hours in PNPP substrate. For peptide epitope mapping, patient serum was diluted 1:100 in 1% BSA/PBS and incubated overnight at 4°C. All samples were assayed in duplicate and averaged.

### Polysaccharide competitive-inhibition ELISA.

Inhibition assays were performed in duplicate as described previously (22). Microtiter plates were coated with 10 μg/mL of D1R and D2R. Sera were diluted 1:250 in 1% BSA in 0.01 M PBS alone or mixed 1:1 with GlcNAc, HIB PRP with methylated human serum albumin, or pneumococcal 23F at 500 μg/mL, incubated at 37°C for 30 minutes and overnight at 4°C. The next day, the inhibition mixture was added to plates coated with D1R or D2R as in the ELISA procedure. Sera diluted 1:1 in PBS served as the 100% activity control.

### Peptides.

Peptides encoding the NT, TM, and EL of D1R and D2R were synthesized by Atlantic Peptides. Peptide sequences and amino acid positions are in Table 2.

### mAbs.

Peripheral blood was obtained from children with acute neuropsychiatric symptoms for PANDAS locally and from the NIMH (25). PBMCs were isolated using a Histopague-1077 (Sigma-Aldrich) gradient, washed, and cultured with pokeweed mitogen in IMDM with 10% AB serum, gentamycin, and penicillin-streptomycin for 7 days to stimulate B cell blast formation. Cells were washed in serum-free IMDM and fused with the K6H6/B5 cell line (CRL 1823, ATCC), as described previously (22, 45). Hybridomas were selected with HAT medium, cloned by limiting dilution (22, 38, 45), and screened with the full-length DR by ELISA. PANDAS-derived human mAb B4C was of the IgG heavy chain, and mAb 3C3.1 and mAb 42.4.1 were of the IgM isotype.

### Immunofluorescence microscopy.

CHO-K1 cells were cultured on Lab-Tek II chamber slides (Thermo Fisher Scientific), transfected with pCDNA-3.1-HA-DRD1 or empty pCDNA3.1, and fixed with 2% paraformaldehyde for 20 minutes at 4°C. Cells were incubated with mAb B4C (1:500) at 37°C for 30 minutes and overnight at 4°C, and then with Alexa Fluor 594–conjugated goat anti–human IgG (Invitrogen) for 1 hour at room temperature. For peptide blocking, B4C was diluted and preincubated with 1 μg/mL D1R EL1 peptide for 2 hours at 37°C before labeling.

### D1R GeneBLAzer signaling assay.

D1R GPCR signaling assays were performed with Tango D1-*bla* USOS cells (Invitrogen). Assays followed the manufacturer’s instructions. Cells were treated with patient sera (1:50), serial dilutions of sera, mAbs, or dopamine in serum-free media and incubated for 5 hours at 37°C and 5% CO_2_. Signaling activity was measured 2 hours after adding the FRET-enabled substrate using a SpectraMax iD3 Microplate Reader (Molecular Devices). Percentage activation was calculated as the ratio of β-lactamase activity to background signal multiplied by 100. For D1R agonist testing, dopamine was serially diluted in assay media 30 minutes before adding patient sera (1:50) following the manufacturer’s protocol.

### cAMP assays.

CHO-K1 cells (ATCC) were transfected with pCDNA-3.1-HA-DRD1 or empty vector pCDNA3.1 one day prior to use for signaling assays (supplied in-house). Cells were maintained in IMDM with 10% FBS (Clonetech). Sequence-verified D1R was obtained from Origene and subcloned into pCDNA3.1+ using primers containing the N-terminal HA epitope tag: Nhe I HA-DRD1 F (TAGCGCTAGCATGTACCCATACGACGTCCCAGACTACGCT) and Kpn I DRD1 R (TAGCGGTACCTCAGGTTGGGTGCTGACCGTTTTG). Transfections were done in 96-well plates with Lipofectamine LTX (Invitrogen) as per the manufacturer’s instructions. Cells were stimulated for 15 minutes with media, sera (1:40), mAbs, or dopamine diluted in media, all containing 0.1% ascorbic acid. cAMP assays with Tango D1-*bla* USOS cells were plated 24 hours prior in a 96-well culture plate (Falcon) in Freestyle Expression Medium. Sera (Cohort 2, 1:50) and PANDAS mAbs (10 ng/mL) were incubated for 20 minutes at 37°C. Cells were lysed and cAMP measured by ELISA using the Parameter cAMP assay (R&D Systems) as per the manufacturer’s instructions.

### D1R-knockdown experiments.

Tango D1-*bla* USOS cells were reverse transfected with Silencer Select siRNA against D1R (Ambion) or nontargeting control siRNA (Invitrogen); 10 nM siRNA-Lipofectamine RNAiMAX (Invitrogen) duplexes were created in Opti-MEM and added to each 384-well plate. Fifteen thousand cells in assay media were added 24 hours prior to performing the D1R GeneBLAzer Signaling Assay with dopamine, sera, or human mAbs. D1R siRNA knockdown efficacy was validated by qRT-PCR. RNA was isolated using TRIzol Reagent (Invitrogen) and DNase-treated total RNA was converted to cDNA using the QuantiTect Reverse Transcription Kit (Qiagen). D1R-specific primers were generated against the D1R CDS sequence provided by Thermo Fisher Scientific and ordered from IDT. cDNA (10 ng) was amplified with D1R primers (0.5 μM) using the QuantiTect Transcription Kit (Qiagen). Data were analyzed with a CFX96 thermocycler (Bio-Rad) using the 2^–ΔΔCt^ method.

### Statistics.

When normality was rejected, we used nonparametric Mann-Whitney *U* tests for pairwise comparisons and Kruskal-Wallis tests for multiple groups, followed by Dunn’s post hoc tests (81). Nonparametric methods were chosen due to non-normal distributions. One-way ANOVA with Tukey’s post hoc tests compared normally distributed means. ROC curves assessed diagnostic test performance using the Wilson-Brown method. Dose-response curves were analyzed using GraphPad Prism’s nonlinear regression. Statistical significance was set at a 2-sided *P* value of less than 0.05, or a more stringent α level when multiple comparisons were considered. Violin plots display data distribution, bar graphs show mean ± SEM, and dot plots mark subgroup medians. The study was powered to detect differences in anti-D1R positivity, with 20 samples/group providing 90% power for a 2-sided 0.01-level χ^2^ test. ROC curves evaluated diagnostic test sensitivity and specificity crucial for clinical use. For more details, see the article on ROC curves (82).

### Data availability.

Data that support and contribute to the conclusions in this paper are provided in the main text, supplemental materials, or the Supporting Data Values file.

## Author contributions

CMM, JZ, and MWC conceived and designed the study. SES and HBP designed and managed the clinical studies and provided samples. CMM, JZ, AK, BR, and MWC developed the methods and reagents for the studies. CMM, JZ, BR, and SR performed the necessary experimentation for the study. CMM, JZ, SES, AK, and MWC analyzed and interpreted the data. CMM and MWC wrote the manuscript. CM, SES, AK, and MWC revised the manuscript. All authors reviewed the manuscript.

## Supplementary Material

Supplemental data

Supporting data values

## Figures and Tables

**Table 1:**
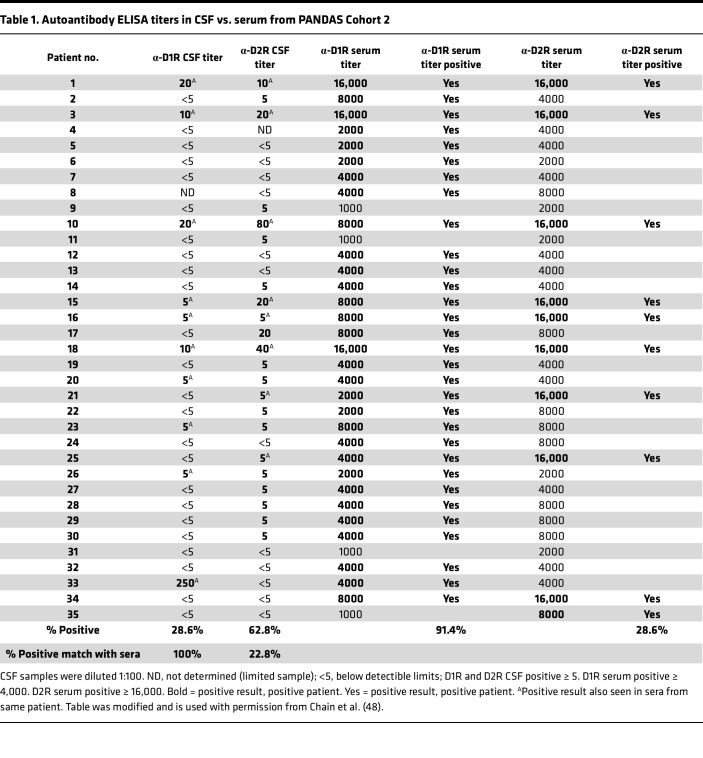
Autoantibody ELISA titers in CSF vs. serum from PANDAS Cohort 2

**Table 2:**
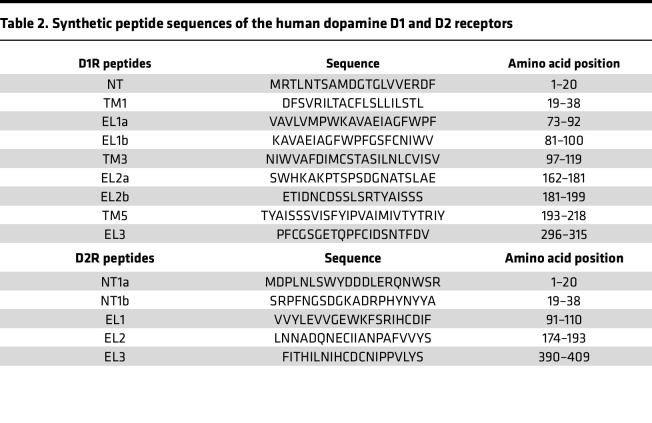
Synthetic peptide sequences of the human dopamine D1 and D2 receptors

## Version 1 09/26/2024

In-Press Preview

## Version 2 11/08/2024

Electronic publication

## References

[1] Orlovska S (2017). Association of streptococcal throat infection with mental disorders: testing key aspects of the PANDAS hypothesis in a nationwide study. JAMA Psychiatry.

[2] Taranta A, Stollerman GH (1956). The relationship of Sydenham’s chorea to infection with group A streptococci. Am J Med.

[3] Platt MP (2020). Th17 lymphocytes drive vascular and neuronal deficits in a mouse model of postinfectious autoimmune encephalitis. Proc Natl Acad Sci U S A.

[4] Fallon BA (2020). Anti-lysoganglioside and other anti-neuronal autoantibodies in post-treatment Lyme disease and Erythema Migrans after repeat infection. Brain Behav Immun Health.

[5] Dale RC (2003). Striatal encephalitis after varicella zoster infection complicated by Tourettism. Mov Disord.

[6] Song E (2021). Divergent and self-reactive immune responses in the CNS of COVID-19 patients with neurological symptoms. Cell Rep Med.

[7] Pavone P (2021). SARS-CoV-2 related paediatric acute-onset neuropsychiatric syndrome. Lancet Child Adolesc Health.

[8] Frankovich J (2015). Five youth with pediatric acute-onset neuropsychiatric syndrome of differing etiologies. J Child Adolesc Psychopharmacol.

[9] Kohler-Forsberg O (2019). A nationwide study in Denmark of the association between treated infections and the subsequent risk of treated mental disorders in children and adolescents. JAMA Psychiatry.

[10] Miyaoka T (2017). Remission of psychosis in treatment-resistant schizophrenia following bone marrow transplantation: a case report. Front Psychiatry.

[11] Mader S (2017). The role of brain-reactive autoantibodies in brain pathology and cognitive impairment. Front Immunol.

[12] Taquet M (2021). 6-month neurological and psychiatric outcomes in 236 379 survivors of COVID-19: a retrospective cohort study using electronic health records. Lancet Psychiatry.

[13] Dale RC (2003). Autoimmunity and the basal ganglia: new insights into old diseases. QJM.

[14] Carapetis JR (2016). Acute rheumatic fever and rheumatic heart disease Nat Rev Dis Primers.

[15] Cunningham MW (2012). Streptococcus and rheumatic fever. Curr Opin Rheumatol.

[16] Swedo SE (1994). Sydenham’s chorea. A model for childhood autoimmune neuropsychiatric disorders. JAMA.

[17] Dale RC, Brilot F (2012). Autoimmune basal ganglia disorders. J Child Neurol.

[18] Dale RC (2012). Antibodies to surface dopamine-2 receptor in autoimmune movement and psychiatric disorders. Brain.

[19] Brimberg L (2012). Behavioral, pharmacological, and immunological abnormalities after streptococcal exposure: a novel rat model of Sydenham chorea and related neuropsychiatric disorders. Neuropsychopharmacology.

[20] Cox CJ (2013). Brain human monoclonal autoantibody from Sydenham chorea targets dopaminergic neurons in transgenic mice and signals dopamine D2 receptor: implications in human disease. J Immunol.

[21] Hoffman KL (2004). A murine model for neuropsychiatric disorders associated with group A beta-hemolytic streptococcal infection. J Neurosci.

[22] Kirvan CA (2003). Mimicry and autoantibody-mediated neuronal cell signaling in Sydenham chorea. Nat Med.

[23] Lotan D (2014). Behavioral and neural effects of intra-striatal infusion of anti-streptococcal antibodies in rats. Brain Behav Immun.

[24] Yaddanapudi K (2010). Passive transfer of streptococcus-induced antibodies reproduces behavioral disturbances in a mouse model of pediatric autoimmune neuropsychiatric disorders associated with streptococcal infection. Mol Psychiatry.

[25] Swedo SE (1998). Pediatric autoimmune neuropsychiatric disorders associated with streptococcal infections: clinical description of the first 50 cases. Am J Psychiatry.

[26] Swedo SE (1997). Identification of children with pediatric autoimmune neuropsychiatric disorders associated with streptococcal infections by a marker associated with rheumatic fever. Am J Psychiatry.

[27] Singer HS (2015). Neuronal antibody biomarkers for Sydenham’s chorea identify a new group of children with chronic recurrent episodic acute exacerbations of tic and obsessive compulsive symptoms following a streptococcal infection. PLoS One.

[28] Kumar A (2015). Evaluation of basal ganglia and thalamic inflammation in children with pediatric autoimmune neuropsychiatric disorders associated with streptococcal infection and tourette syndrome: a positron emission tomographic (PET) study using 11C-[R]-PK11195. J Child Neurol.

[29] Pollak TA (2020). Autoimmune psychosis: an international consensus on an approach to the diagnosis and management of psychosis of suspected autoimmune origin. Lancet Psychiatry.

[30] Mohammad SS, Dale RC (2018). Principles and approaches to the treatment of immune-mediated movement disorders. Eur J Paediatr Neurol.

[31] Frick LR (2018). Differential binding of antibodies in PANDAS patients to cholinergic interneurons in the striatum. Brain Behav Immun.

[32] Pilli D (2020). Pro-inflammatory dopamine-2 receptor-specific T cells in paediatric movement and psychiatric disorders. Clin Transl Immunology.

[33] Kirvan CA (2006). Streptococcal mimicry and antibody-mediated cell signaling in the pathogenesis of Sydenham’s chorea. Autoimmunity.

[34] Lotan D (2014). Antibiotic treatment attenuates behavioral and neurochemical changes induced by exposure of rats to group a streptococcal antigen. PLoS One.

[35] Goldstein I (1967). Immunological relationship between Streptococcus A polysaccharide and the structural glycoproteins of heart valve. Nature.

[36] Ben-Pazi H (2013). Dopamine receptor autoantibodies correlate with symptoms in Sydenham’s chorea. PLoS One.

[37] Williams KA (2016). Randomized, controlled trial of intravenous immunoglobulin for pediatric autoimmune neuropsychiatric disorders associated with streptococcal infections. J Am Acad Child Adolesc Psychiatry.

[38] Kirvan CA (2006). Antibody-mediated neuronal cell signaling in behavior and movement disorders. J Neuroimmunol.

[39] Swedo SE (2012). From research subgroup to clinical syndrome: modifying the PANDAS criteria to describe PANS (pediatric acute-onset neuropsychiatric syndrome). Pediatr Ther.

[41] Ben-Pazi H (2013). Analysis of transduction efficiency, tropism and axonal transport of AAV serotypes 1, 2, 5, 6, 8 and 9 in the mouse brain. PLoS One.

[42] Shikhman AR (1994). Cytokeratin peptide SFGSGFGGGY mimics N-acetyl-beta-D-glucosamine in reaction with antibodies and lectins, and induces in vivo anti-carbohydrate antibody response. J Immunol.

[43] McCarty M (1956). Variation in the group-specific carbohydrate of group A streptococci. II. Studies on the chemical basis for serological specificity of the carbohydrates. J Exp Med.

[44] Dudding BA, Ayoub EM (1968). Persistence of streptococcal group A antibody in patients with rheumatic valvular disease. J Exp Med.

[45] Shikhman AR, Cunningham MW (1994). Immunological mimicry between N-acetyl-beta-D-glucosamine and cytokeratin peptides. Evidence for a microbially driven anti-keratin antibody response. J Immunol.

[46] Van Gastel J (2018). β-arrestin based receptor signaling paradigms: potential therapeutic targets for complex age-related disorders. Front Pharmacol.

[47] Cox CJ (2015). Antineuronal antibodies in a heterogeneous group of youth and young adults with tics and obsessive-compulsive disorder. J Child Adolesc Psychopharmacol.

[48] Chain J (2020). Autoantibody biomarkers for basal ganglia encephalitis in Sydenham chorea and pediatric autoimmune neuropsychiatric disorder associated with streptococcal infections. Front Psychiatry.

[49] Shimasaki C (2020). Evaluation of the Cunningham Panel™ in pediatric autoimmune neuropsychiatric disorder associated with streptococcal infection (PANDAS) and pediatric acute-onset neuropsychiatric syndrome (PANS): changes in antineuronal antibody titers parallel changes in patient symptoms. J Neuroimmunol.

[50] Kirvan CA (2023). IgG2 rules: N-acetyl-beta-D-glucosamine- specific IgG2 and Th17/Th1 cooperation may promote the pathogenesis of acute rheumatic heart disease and be a biomarker of the autoimmune sequelae of Streptococcus pyogenes. Front Cardiovasc Med.

[51] Li Y (2006). Mimicry and antibody-mediated cell signaling in autoimmune myocarditis. J Immunol.

[52] Li H (2014). Autoimmune basis for postural tachycardia syndrome. J Am Heart Assoc.

[53] Yu X (2009). Development of cardiomyopathy and atrial tachyarrhythmias associated with activating autoantibodies to beta-adrenergic and muscarinic receptors. J Am Soc Hypertens.

[54] Shikhman AR (1993). A subset of mouse monoclonal antibodies cross-reactive with cytoskeletal proteins and group A streptococcal M proteins recognizes N-acetyl-beta-D-glucosamine. J Immunol.

[55] Mohammad SS (2014). Herpes simplex encephalitis relapse with chorea is associated with autoantibodies to N-methyl-D-aspartate receptor or dopamine-2 receptor. Mov Disord.

[56] Gorton D (2016). Repeat exposure to group A streptococcal M protein exacerbates cardiac damage in a rat model of rheumatic heart disease. Autoimmunity.

[57] Cunningham MW, Cox CJ (2016). Autoimmunity against dopamine receptors in neuropsychiatric and movement disorders: a review of Sydenham chorea and beyond. Acta Physiol (Oxf).

[58] Borda E (1984). A circulating IgG in Chagas’ disease which binds to beta-adrenoceptors of myocardium and modulates their activity. Clin Exp Immunol.

[59] Stavrakis S (2009). Activating autoantibodies to the beta-1 adrenergic and m2 muscarinic receptors facilitate atrial fibrillation in patients with Graves’ hyperthyroidism. J Am Coll Cardiol.

[60] Quinn A (1998). Immunological relationship between the class I epitope of streptococcal M protein and myosin. Infect Immun.

[61] McNamara C (2008). Coiled-coil irregularities and instabilities in group A Streptococcus M1 are required for virulence. Science.

[62] Yilmaz S, Mink JW (2020). Treatment of chorea in childhood. Pediatr Neurol.

[63] Wallukat G, Schimke I (2014). Agonistic autoantibodies directed against G-protein-coupled receptors and their relationship to cardiovascular diseases. Semin Immunopathol.

[64] Hohberger B (2021). Inhibitory and agonistic autoantibodies directed against the β_2_-adrenergic receptor in pseudoexfoliation syndrome and glaucoma. Front Neurosci.

[65] Jahns R (2004). Direct evidence for a beta 1-adrenergic receptor-directed autoimmune attack as a cause of idiopathic dilated cardiomyopathy. J Clin Invest.

[66] Bien CG (2012). Immunopathology of autoantibody-associated encephalitides: clues for pathogenesis. Brain.

[67] Rafeek RAM (2021). Group A streptococcal antigen exposed rat model to investigate neurobehavioral and cardiac complications associated with post-streptococcal autoimmune sequelae. Animal Model Exp Med.

[68] Xu J (2021). Antibodies from children with PANDAS bind specifically to striatal cholinergic interneurons and alter their activity. Am J Psychiatry.

[69] Dileepan T (2016). Group A Streptococcus intranasal infection promotes CNS infiltration by streptococcal-specific Th17 cells. J Clin Invest.

[70] Frankovich J (2017). Clinical management of pediatric acute-onset neuropsychiatric syndrome: part II-use of immunomodulatory therapies. J Child Adolesc Psychopharmacol.

[71] Perlmutter SJ (1999). Therapeutic plasma exchange and intravenous immunoglobulin for obsessive-compulsive disorder and tic disorders in childhood. Lancet.

[72] Garvey MA (2005). Treatment of Sydenham’s chorea with intravenous immunoglobulin, plasma exchange, or prednisone. J Child Neurol.

[73] Walker K (2012). Treatment of Sydenham chorea with intravenous immunoglobulin. J Child Neurol.

[74] Kirvan CA (2007). Tubulin is a neuronal target of autoantibodies in Sydenham’s chorea. J Immunol.

[75] Mertens NM (2000). Molecular analysis of cross-reactive anti-myosin/anti-streptococcal mouse monoclonal antibodies. Mol Immunol.

[76] Adderson EE (1998). Molecular analysis of polyreactive monoclonal antibodies from rheumatic carditis: human anti-N-acetyl- glucosamine/anti-myosin antibody V region genes. J Immunol.

[77] Quinn A (1995). Autoantibody germ-line gene segment encodes VH and VL regions of a human anti-streptococcal monoclonal antibody recognizing streptococcal M protein and human cardiac myosin epitopes. J Immunol.

[78] Antone SM (1997). Molecular analysis of V gene sequences encoding cytotoxic anti-streptococcal/ anti-myosin monoclonal antibody 36.2.2 that recognizes the heart cell surface protein laminin. J Immunol.

[79] Dan JM (2019). Recurrent group A Streptococcus tonsillitis is an immunosusceptibility disease involving antibody deficiency and aberrant T_FH_ cells. Sci Transl Med.

[81] Hart A (2001). Mann-Whitney test is not just a test of medians: differences in spread can be important. BMJ.

[82] Nahm FS (2022). Receiver operating characteristic curve: overview and practical use for clinicians. Korean J Anesthesiol.

