# Supplementary material for: Dopamine receptor autoantibody signaling in infectious sequelae differentiates movement versus neuropsychiatric disorders
Source: JCI Insight. 2024 Nov 8;9(21):e164762. doi: 10.1172/jci.insight.164762 (PMC11601707; doi:10.1172/jci.insight.164762)
Supplement: Supplemental data [file jciinsight-9-164762-s243.pdf]

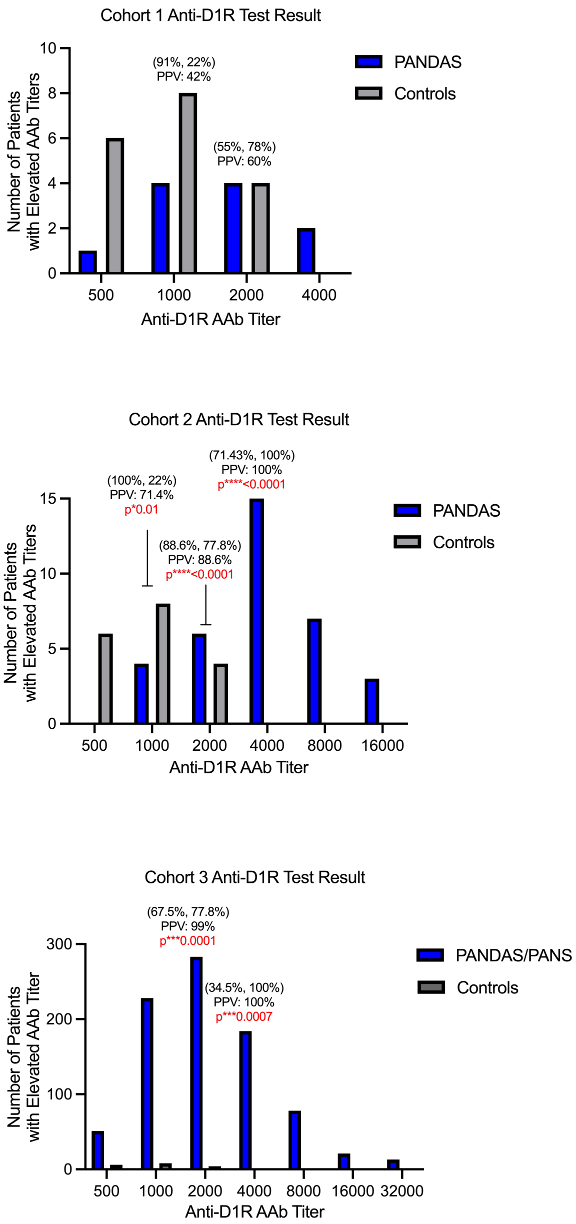

Supplemental Figure 1. PANDAS Patient D1R AAb titer distributions and relevant sensitivity, specificity, and positive predictive rates. Contingency data from ROC curve analysis (Figure 1) were used for each cutoff value, comparing positive test/disease versus negative test/no disease. Fisher's exact test was used to determine significance (P values shown). Sensitivity, specificity, and positive predictive values for D1R AAb titers at each titer are presented.

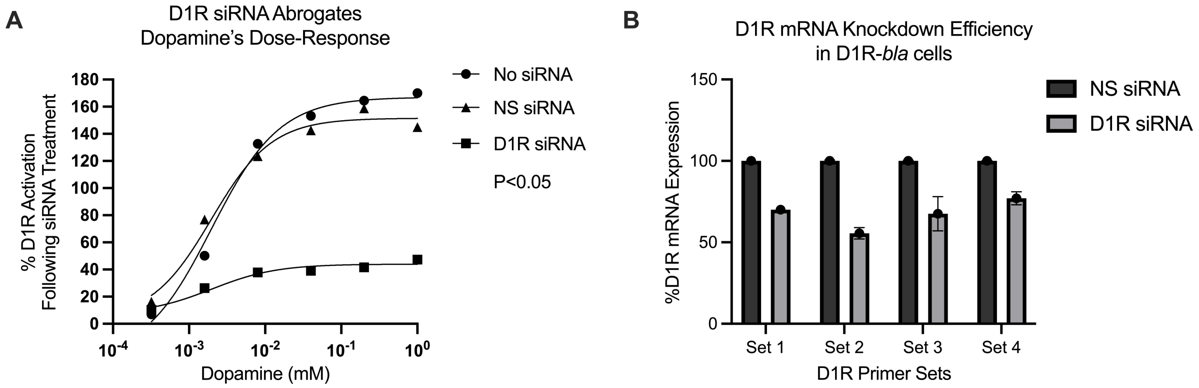

Supplemental Figure 2. D1R specific siRNA knockdown efficiency. A) D1R specific siRNA (D1R siRNA) reduces the dopamine dose-response (square dots) in the Tango D1R GeneBLAzer Assay following siRNA transfection of D1R-*bla* U20S cells when compared to the nonspecific siRNA (NS siRNA, triangles) and no siRNA control (black circles). *P<0.05, nonlinear regression extra-sum-of-squares F test. No difference between the NS siRNA and the non-treated control (circle dots). **B)** RT-qPCR D1R mRNA knockdown efficiency following treatment with D1R or NS siRNA with four distinct D1R primer sets (outlined in the methods).

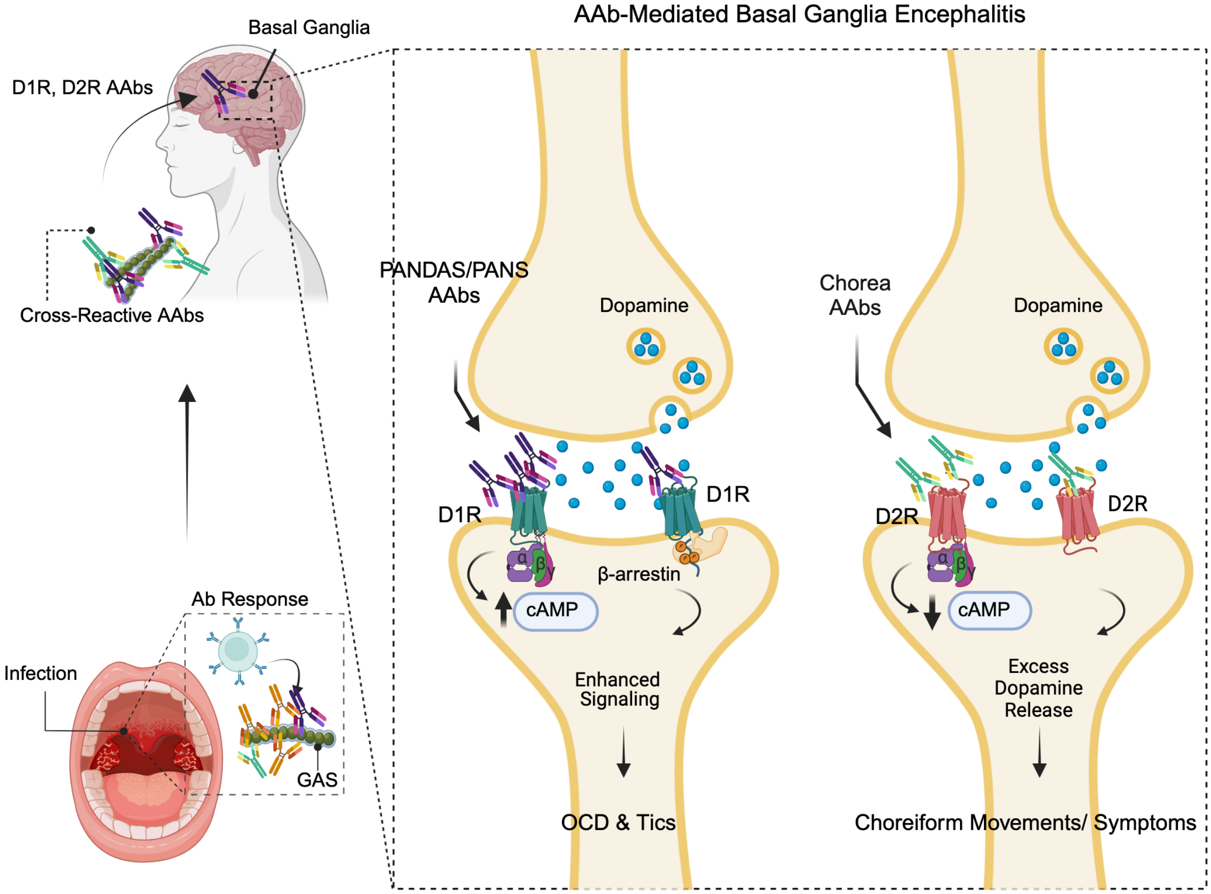

Supplemental Figure 3. D1R autoantibody-mediated pathogenesis in the neuropsychiatric sequelae vs D2R in choreatic movement disorders. D1R AAbs in neuropsychiatric sequelae that predominantly manifest as OCD and/or tics (PANDAS/PANS) activate D1R, increase intracellular cAMP and β-arrestin activity, and enhance the neurotransmission of dopamine to impact downstream signaling. While D2R AAbs from SC decrease cAMP and increase dopamine release in which excess dopamine accumulation correlates with the choreiform movement phenotype in SC and cases of PANDAS mixed with choreatic movements. The pathogenesis derives from the hypothesis that 1) elevated D2R AAbs lead to excess dopamine affecting not only choreiform movements but also D1R which 2) may bind dopamine more avidly or allosterically due to the presence of D1R AAbs which then 3) sensitize the D1R to excess dopamine signaling to produce a more exaggerated response leading to neuropsychiatric symptoms.
